# Rationale for Stereotactic Body Radiation Therapy in Treating Patients with Oligometastatic Hormone-Naïve Prostate Cancer

**DOI:** 10.3389/fonc.2013.00293

**Published:** 2013-12-03

**Authors:** Onita Bhattasali, Leonard N. Chen, Michael Tong, Siyuan Lei, Brian T. Collins, Pranay Krishnan, Christopher Kalhorn, John H. Lynch, Simeng Suy, Anatoly Dritschilo, Nancy A. Dawson, Sean P. Collins

**Affiliations:** ^1^Department of Radiation Medicine, Georgetown University Hospital, Washington, DC, USA; ^2^Department of Radiology, Georgetown University Hospital, Washington, DC, USA; ^3^Department of Neurosurgery, Georgetown University Medical Center, Washington, DC, USA; ^4^Department of Urology, Georgetown University Hospital, Washington, DC, USA; ^5^Department of Oncology, Lombardi Comprehensive Cancer Center, Georgetown University Medical Center, Washington, DC, USA

**Keywords:** prostate cancer, SBRT, IGRT, cyberknife, oligometastases, hormone-naïve

## Abstract

Despite advances in treatment for metastatic prostate cancer, patients eventually progress to castrate-resistant disease and ultimately succumb to their cancer. Androgen deprivation therapy (ADT) is the standard treatment for metastatic prostate cancer and has been shown to improve median time to progression and median survival time. Research suggests that castrate-resistant clones may be present early in the disease process prior to the initiation of ADT. These clones are not susceptible to ADT and may even flourish when androgen-responsive clones are depleted. Stereotactic body radiation therapy (SBRT) is a safe and efficacious method of treating clinically localized prostate cancer and metastases. In patients with a limited number of metastatic sites, SBRT may have a role in eliminating castrate-resistant clones and possibly delaying progression to castrate-resistant disease.

## Stereotactic Body Radiation Therapy

**Figure 1:**
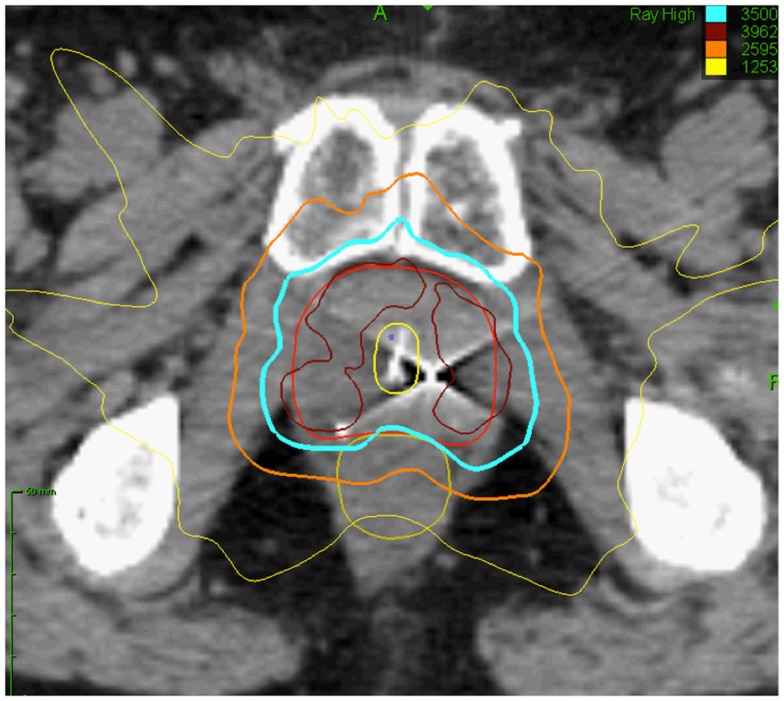
**Prostate SBRT: treatment planning axial computed tomography images demonstrating the prostate (red line), prostatic urethra (yellow), and rectum (green line)**. Isodose lines shown as follows: 115% of the prescription dose, maroon line; 100% of the prescription dose, light blue line: 75% of the prescription dose, orange line; and 35% of the prescription dose, green line.

Radiation oncologists strive to maximize tumor control while minimizing normal tissue toxicity. Over the past several years, advances in image-guided radiation treatment (IGRT) have allowed the treatment of tumors with increased efficacy and reduced toxicity (1–4). For example, stereotactic body radiation therapy (SBRT) may improve tumor control and reduce treatment-related toxicity through improved targeting and management of tumor motion (5). Accurate tumor targeting means that radiation may be delivered with relatively narrow margins to account for uncertainty in target position. This allows for high-dose, extremely hypofractionated treatment courses (1–5 fractions) that may be more radiobiologically effective and are certainly more convenient for patients (6, 7). For example, the CyberKnife Radiosurgical System (Accuray) is capable of localizing the prostate and adjusting the radiation beam accordingly in real time throughout a treatment fraction (8). This feature allows for a reduction in the planning target volume (PTV) and therefore better limits the dose to adjacent rectum and bladder (Figure 1). Multi-institutional experience demonstrates that this technology allows investigators to administer higher doses to the prostate with biochemical disease-free survival and toxicity rates similar to conventional treatments (9–14). It is hoped that SBRT will also positively impact patient outcomes in patients with limited metastatic disease.

## Oligometastases

**Figure 2:**
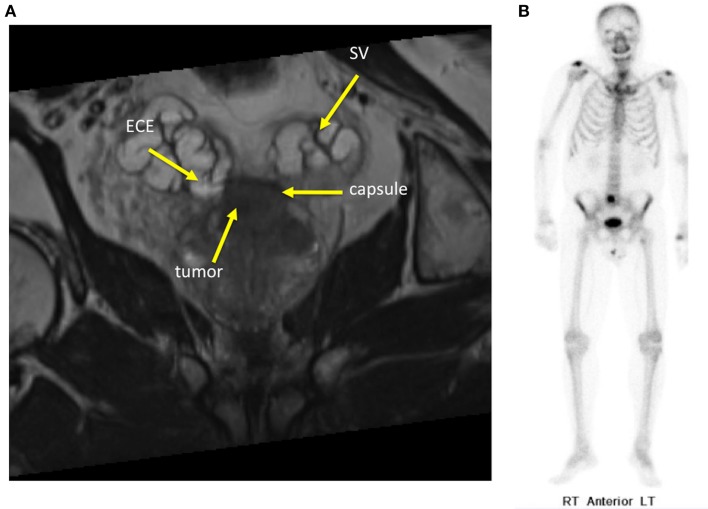
**Sixty-year-old gentleman with oligometastatic prostate cancer**. He presented with back pain and his PSA was 35 ng/ml. DRE was abnormal and imaging revealed: **(A)** Coronal T2-weighted multiplanar reconstruction MRI image demonstrating extracapsular extension into the seminal vesicles. **(B)** Bone scan demonstrating a solitary L5 vertebral body metastasis.

Patients with controlled primaries and “oligometastatic” disease may experience long-term stability in the number of metastatic sites (15). Oligometastatic prostate cancer has been defined as five or fewer sites due to the more favorable outcomes seen in these patients (Figure 2) (16). Hellman and Weichselbaum first proposed the existence of oligometastatic disease as a clinically significant state separate from polymetastatic disease and suggested a more causal relationship between the size or grade of a tumor and its propensity for metastatic spread (17). Corbin et al. expanded on this concept suggesting the development of a specific oligometastatic phenotype over the natural course of a cancer’s evolution that is less aggressive than other metastatic phenotypes (18). This theory has been corroborated by microRNA analysis of clinically limited metastatic disease that accurately characterizes which patients will remain oligometastatic and which patients will proceed to polymetastatic disease (19). For patients with limited metastatic sites, SBRT to the oligometastases may offer long-term disease control and impact survival (20). Data are emerging that patients with limited asymptomatic metastases may experience improved disease-free survival and quality of life after SBRT (21). We hypothesize that in oligometastatic prostate cancer patients, androgen deprivation therapy (ADT) would eliminate micrometastatic disease while SBRT would eradicate large tumor deposits that may be more likely to develop castrate-resistant clones.

## Androgen Deprivation Therapy for Metastatic Prostate Cancer

The current treatment for newly diagnosed metastatic prostate cancer is hormone ablation via luteinizing hormone-releasing hormone (LHRH) analog until disease progression (22). The response rate for primary hormonal therapy for men with metastatic prostate cancer exceeds 80% and the median duration of response is approximately 18–24 months (22). Patients with high volume metastatic disease have a poorer prognosis with a median time to prostate-specific antigen (PSA) progression of about only 10 months and median time to clinical progression (e.g., worsening bone metastases) of about 14 months (23). In contrast, patients with low volume metastatic disease have a 22-month median time to PSA progression with androgen ablation alone and median time to clinical progression of more than 3 years (23). The median overall survival for men commencing androgen ablation with clinically evident metastatic disease is about 30 months (22). Survival varies depending on the extent of disease and location of the bone metastases (16, 23–27). All patients will ultimately progress despite the initial success of this approach. Castrate-resistant prostate cancer (CRPC) remains an incurable disease resulting in considerable morbidity. Alternative hormonal agents or chemotherapy may be employed at the time of castrate resistance and provide small overall survival benefits (28).

## Chemotherapeutic Agents for Castrate-Resistant Prostate Cancer

Early investigation of chemotherapeutic agents for metastatic CRPC showed that mitoxantrone combined with prednisone improved pain and quality of life when compared to prednisone alone (29, 30). Unfortunately, mitoxantrone did not prolong survival in randomized trials (31, 32). Docetaxel was the first chemotherapeutic agent able to demonstrate increased survival in metastatic CRPC in addition to decreased pain and improved quality of life (33). Median survival increased by 2.9 months in the cohort who received docetaxel compared to mitoxantrone.

The breakthrough with docetaxel has led to subsequent advances in systemic therapy for metastatic prostate cancer. Multiple hormonal and non-hormonal agents have emerged in recent phase III clinical trials that demonstrate increased overall survival time (outlined in Tables 1 and 2) (34–39). Hormonal agents target adrenal testosterone production that is shielded from conventional ADT. Abiraterone inhibits androgen production by blocking enzymes crucial to testosterone synthesis (34). Enzalutamide does not lower intratumoral testosterone but is a potent androgen receptor antagonist that acts by blocking androgen activity within cancer cells (36). Novel non-hormonal agents have also been efficacious in the setting of CRPC. Sipuleucel-T is a therapeutic cancer vaccine that acts as an immunostimulant specifically targeting the prostatic acid phosphatase (PAP) antigen found on prostate cancer cells (37). Radium-223 is a radiopharmaceutical agent that targets bony tissue and destroys metastatic prostate cancer cells through alpha particle emission (38). Additional phase III trials with newer agents are underway. To date, no single agent has demonstrated a PSA response rate greater than 54% or an overall survival benefit greater than 5 months, and further innovation through new agents or combination regimens is necessary to optimize survival.

**Table 1:** **Prostate-specific antigen response rate of new chemotherapeutic agents for metastatic CRPC**.

Trial	Treatment group	Drug class	Mechanism of action	Control group	Treatment group response rate (%)	Control group response rate (%)	*P*-value
TAX 327	Docetaxel + prednisone	Taxoid	Microtubule disassembly inhibitor	Mitoxantrone + prednisone	45	32	<0.001
TROPIC	Cabazitaxel + prednisone	Taxoid	Microtubule disassembly inhibitor	Mitoxantrone + prednisone	39.2	17.8	=0.0002
COU-AA301	Abiraterone + prednisone	Hormonal agent	Cytochrome P4S0 17A1 inhibitor	Placebo + prednisone	29	6	<0.001
AFFIRM	En2alutamide	Hormonal agent	Androgen receptor antagonist	Placebo	54	2	<0.001
IMPACT	Sipuleucel-T	Cancer vaccine	PA2024 activated peripheral-mononuclear cells	Placebo	2.6	1.3	Not significant
ALSYMPCA	Radium-223	Radio pharmaceutical	Bone-targeted alpha radiation	Placebo	16	6	<0.001

**Table 2:** **Overall survival benefit of new chemotherapeutic agents for metastatic CRPC**.

Trial	Patients	Treatment group	Control group	Median improvement in overall survival (months)	*P*-value
TAX 327	1006	Docetaxel + prednisone	Mitoxantrone + prednisone	2.9	=0.004
TROPIC	755	Cabazitaxel + prednisone	Mitoxantrone + prednisone	2.4	<0.0001
COU-AA-301	119S	Abiraterone + prednisone	Placebo + prednisone	3.9	<0.001
AFFIRM	1199	Enzalutamide	Placebo	4.8	<0.0001
IMPACT	512	Sipuleucel-T	Placebo	4.1	=0.03
ALSYMPCA	922	Radium-223	Placebo	3.6	<0.001

## Rationale for Treatment of the Prostate in the Presence of Oligometastatic Disease

We believe an effective radiotherapeutic approach in the prostate may improve long-term outcomes with limited toxicity in patients with oligometastatic disease. The addition of prostate radiotherapy to ADT has been shown to significantly improve progression-free survival and overall survival with acceptable morbidity in patients with locally advanced prostate cancer (40, 41). While a slight increase in overall bother from urinary and bowel symptoms may occur from combined therapy, the difference is minimal and does not meet the threshold for clinical significance (42). The SPCG-7/SFUO-3 trial for patients with locally advanced prostate cancer achieved a 12% reduction in 10-year prostate cancer specific mortality when radiotherapy was combined with endocrine treatment (41). The trial observed a 10-year overall survival benefit of 8.9% consistent with a 7-year overall survival benefit of 8% with the addition of radiation therapy in the NCIC CTG PR.3/MRC UK PR07 trial (40, 41).

**Figure 3:**
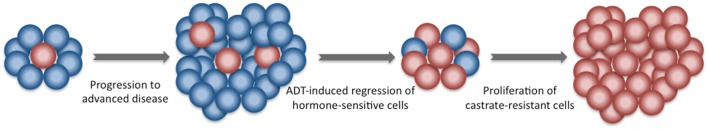
**Development of castrate-resistant prostate cancer**. Newly diagnosed prostate cancer is composed of a group of heterogeneous cells. The majority is hormone-sensitive. A minority are castration-resistant. Following the initiation of ADT, castration-resistant cells have a survival advantage and give rise to a more aggressive castration-resistant prostate cancer.

The mechanism of such benefit is currently unclear. Castrate-resistant clones may be present in the prostate prior to the initiation of ADT and they could be enriched through clonal selection after testosterone decline (Figure 3) (43). Animal models support the use of early local treatment to eliminate androgen-independent clones (44, 45). Radiotherapy, which eradicates androgen-sensitive and androgen insensitive clones with similar efficacy, may be effective at eradicating androgen-independent clones. This has the potential to delay the time to castrate resistance and hence prolong disease control.

Studies in which routine post-radiotherapy prostate biopsies have been performed following primary ADT reveal a high rate of persistence of local disease (46). In the SPCG-7 trial, the post-radiation therapy biopsy positivity rate was an unacceptable 66% (46). Local control is important in this malignancy, as problems resulting from uncontrolled local disease are significant including urinary obstruction (47). Palliative transurethral resection of the prostate (TURP) and/or radiation therapy may be less effective than primary treatment when the disease burden is lower (48, 49). It is also evident that local failures can lead to a second wave of distant metastases (50). Achieving improved local control within the prostate therefore carries promise of reducing the sequelae attributable to uncontrolled local disease as well as the prevention of new metastases.

Prostate cancer growth is dependent on androgen activation of androgen receptors. ADT decreases testicular androgens. Although testes are the major source of testosterone in normal men, the intratumoral synthesis of testosterone from weak adrenal androgens appears to be a substantial source of intraprostatic androgen following ADT (51). Intraprostatic androgen synthesis may protect primary prostate cancer cells from ADT and provide a sanctuary for prostate cancer cells to progress to castrate resistance. We propose that SBRT may eliminate this sanctuary delaying the emergence of castrate resistance.

## Rationale for Treatment of Bone Oligometastases

Prostate cancer has a tropism for bone, making it the most common, and frequently the only, site of metastatic disease (52–54). Greater than 80% of men with metastatic prostate cancer have radiographic evidence of bone involvement. Skeletal complications are a major cause of morbidity in men with prostate cancer. Early in the natural history of the disease, bone metastases are generally asymptomatic, but ultimately at least 40% of patients will be affected by bone pain, 20% will experience a pathologic fracture, and 5% will develop a spinal cord compression. Collectively, skeletal metastases can lead to decreased performance status and devastating neurologic injury. Bone-targeted therapy, such as zoledronic acid and denosumab, decrease but do not eliminate the morbidity associated with bone lesions (55–57). Radiation therapy is typically reserved for symptomatic disease, when the burden of disease is greater and morbidity such as fracture may not be avoidable. Delaying radiation therapy to this point might limit its efficacy in reducing bone morbidity.

Recent reports have suggested that SBRT is safe and effective in treating bone lesions involving long bones and the spinal column (58, 59). Earlier studies administered hypofractionated regimens more similar to conventional radiotherapy delivery with doses of 50 Gy in 10 fractions (21). Several questions remain given the lack of long-term data compared to more conventional radiation therapy. No optimal SBRT regimen has been established due to the variation in target volume and proximity to normal structures (60). However, SBRT has been administered up to 48 Gy in 3 fractions to multiple metastatic sites simultaneously, and results have shown promising long-term disease control with minimal grade 3+ toxicity (61). The potential benefits of combining radiation with systemic agents has also been demonstrated (62, 63).

Patients treated with SBRT at oligometastatic sites have demonstrated excellent outcomes. Among a cohort of patients with oligometastatic disease and detectable PSA, 100% achieved local control with SBRT to the metastatic lesions, and over half the patients achieved an undetectable or declining PSA by a median follow up of 4.8 months (64). In another study of men with oligometastases following prostate treatment, salvage SBRT deferred initiation of ADT with a 2-year local control rate of 100% and a clinical progression-free survival of 42% (65). Neither study observed grade 3+ toxicity. Larger studies with more homogeneous patient populations are required to define the potential benefits of SBRT in the setting of prostate cancer. In addition, further research is needed to determine the potential impact of SBRT on systemic disease when combined with immunostimulating agents such as sipuleucel-T (66).

**Figure 4:**
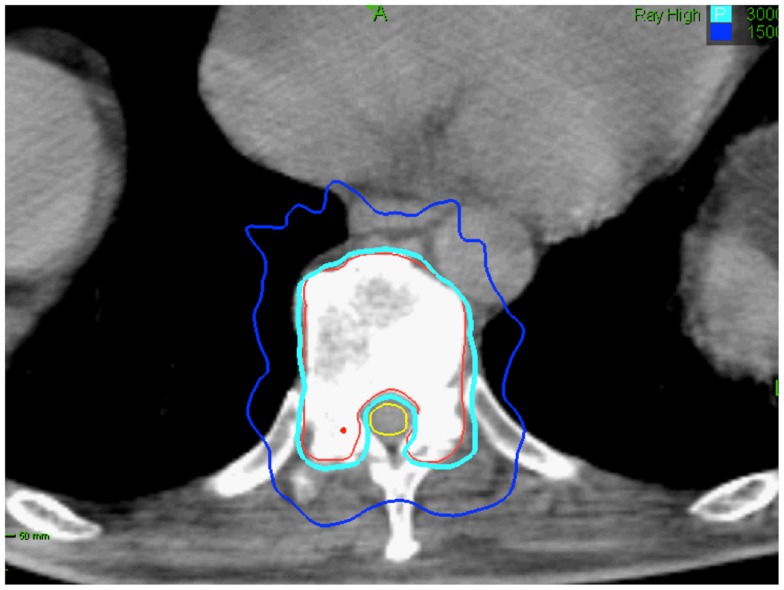
**Seventy-five-year-old gentleman with oligometastatic prostate cancer and a T11 vertebral body metastasis**. The decision was to proceed with ADT and SBRT. ADT was initiated. The vertebral body was treated with 30 Gy in 5 fractions. Treatment planning axial computed tomography images demonstrating the GTV (red) and spinal cord (yellow). Isodose lines shown as follows: 100% of the prescription dose (light blue line) and 50% of the prescription dose (dark blue line). The maximum point to the spinal cord and esophagus were 30 and 35 Gy, respectively (69).

Limited data exist on how radiation dose and fractionation affect the risk of fracture following radiation therapy. Pathologic vertebral body fractures have been described in patients treated with SBRT. They are more common when the lesion is lytic and ≥20% of the vertebral body is involved (67). Vertebral body fracture progression may occur in 40% of vertebrae treated with single-dose SBRT (67). Treating patients early in the disease course to decrease the extent of bone/vertebral body involvement at the time of SBRT treatment and fractionation may reduce the likelihood of normal bone injury (Figure 4) (68).

## Treatment Toxicity and Quality of Life

We hypothesize that SBRT will decrease tumor burden in the prostate and bone and hence improve long-term well-being. However, if SBRT to the prostate and oligometastases caused a significant rate of high-grade late toxicity and/or adversely affected patients’ long-term quality of life this approach would not be worth pursuing further. Prostate SBRT may cause urinary and rectal injury while bone SBRT may promote fractures. The severity and duration of these toxicities varies among patients and has never been prospectively assessed in this patient population. Patients receiving primary ADT have a quality of life that is indistinguishable from a matched normal male population and a quality of life significantly better than that of men with castrate-resistant disease (70). Our experience suggests that prostate SBRT will not adversely affect this (71).

## Conclusion

Castrate-resistant prostate cancer remains a complex and incurable disease. ADT is successful in delaying the progression to castrate-resistant disease and improving overall survival. Unfortunately, castrate-resistant clones may be present early in the disease process even prior to initiation of ADT, creating the need for alternative treatments. Several chemotherapeutic agents have been developed to treat metastatic prostate cancer, but the benefits of these drugs have been small to date. Radiation therapy is effective for treating bone metastases but is typically reserved for late-stage, symptomatic disease. SBRT has been demonstrated as a safe and efficacious modality for bone lesions. Implementation of SBRT early in the disease process may decrease the morbidity associated with bone lesions and reduce overall tumor burden, in turn delaying progression of disease and improving both the quality and length of life.

## Author Contributions

Onita Bhattasali and Leonard N. Chen are lead authors who participated in manuscript drafting, table/figure creation, and manuscript revision. Michael Tong aided in table/figure creation. Siyuan Lei is the dosimetrist who contributed dosimetric data and figures. Pranay Krishnan aided in figure creation. Anatoly Dritschilo, Christopher Kalhorn, Simeng Suy, Brian T. Collins, John H. Lynch, and Nancy A. Dawson are senior authors who aided in drafting the manuscript and manuscript revision. Sean P. Collins is the corresponding author who initially developed the concept, and drafted and revised the manuscript. All authors read and approved the final manuscript.

## Conflict of Interest Statement

Sean P. Collins and Brian T. Collins serve as clinical consultants to Accuray Inc. The other authors declare that they have no competing interests.

## Abbreviations

ADT, androgen deprivation therapy; CRPC, castrate-resistant prostate cancer; DVH, dose-volume histogram; GTV, gross target volume; IGRT, image-guided radiation treatment; LHRH, luteinizing hormone-releasing hormone; PAP, prostatic acid phosphatase; PSA. prostate-specific antigen; PTV, planning target volume; SBRT, stereotactic body radiation therapy; TURP, transurethral resection of the prostate.
